# Examination of the TIGIT, CD226, CD112, and CD155 Immune Checkpoint Molecules in Peripheral Blood Mononuclear Cells in Women Diagnosed with Early-Onset Preeclampsia

**DOI:** 10.3390/biomedicines9111608

**Published:** 2021-11-03

**Authors:** Laszlo Szereday, David U. Nagy, Beata Csiszar, Dora Kevey, Timoteus Feik, Matyas Meggyes

**Affiliations:** 1 Department of Medical Microbiology and Immunology, Medical School, University of Pecs, 12 Szigeti Street, 7624 Pecs, Hungary; feik.timo@gmail.com (T.F.); meggyes.matyas@pte.hu (M.M.); 2 Janos Szentagothai Research Centre, 20 Ifjusag Street, 7624 Pecs, Hungary; 3 Institute of Geobotany/Plant Ecology, Martin-Luther-University, Große Steinstraße 79/80, D-06108 Halle (Saale), Germany; davenagy9@gmail.com; 4 1st Department of Medicine, Medical School, University of Pecs, 13 Ifjusag Street, 7624 Pecs, Hungary; csiszar.beata@pte.hu; 5 Department of Obstetrics and Gynaecology, Medical School, University of Pecs, 17 Edesanyak Street, 7624 Pecs, Hungary; keveydorka@gmail.com

**Keywords:** preeclampsia, immune checkpoint, TIGIT, CD226, CD112, CD155

## Abstract

Early-onset preeclampsia is a common obstetrical disease with a potential genetic background and is characterized by the predominance of Th1 immune response. However, although many studies investigated the immunological environment in preeclamptic patients, no information is available about the potential role of the TIGIT/CD226/CD112/CD155 immune checkpoint pathway. A total of 37 pregnant women diagnosed with early-onset preeclampsia and 36 control women with appropriately matched gestational age were enrolled in this study. From venous blood, mononuclear cells were isolated and stored in the freezer. Using multicolor flow cytometry T-, NK cell and monocyte subpopulations were determined. After characterization of the immune cell subsets, TIGIT, CD226, CD112, and CD155 surface expression and intracellular granzyme B content were determined by flow cytometer. Significantly decreased CD226 expression and increased CD112 and CD155 surface expression were detected in almost all investigated T-cell, NK cell, and monocyte subpopulations in women diagnosed with preeclampsia compared to the healthy group. Furthermore, reduced TIGIT and granzyme B expression were measured only in preeclamptic CD8+ T cells compared to healthy pregnant women. A decreased level of the activatory receptor CD226 in effector lymphocytes accompanied with an elevated surface presence of the CD112 and CD155 ligands in monocytes could promote the TIGIT/CD112 and/or TIGIT/CD155 ligation, which mediates inhibitory signals. We assume that the inhibition of the immune response via this immune checkpoint pathway might contribute to compensate for the Th1 predominance during early-onset preeclampsia.

## 1. Introduction

Preeclampsia (PE) is a severe pregnancy-related complication that occurs in 3–8% of pregnancies. The incidence of the disease is increasing, especially in developing countries, and without proper health care can lead to preterm birth accompanied by fetal growth restriction [1]. The etiology of the disease is still unknown, but the overreaction of the expected maternal inflammation could lead to a restricted migration of the cytotrophoblast with endothelial injury. These processes can induce insufficient implantation and result from a small-sized placenta [2], which try to decompensate continuously when fetal growth is accelerated and leads to the appearance of the maternal symptoms [1]. Preeclampsia is characterized by maternal symptoms, which is, according to the International Society for the Study of Hypertension in Pregnancy (ISSHP), gestational hypertension (≥140/90 mmHg on at least two occasions measured 4 h apart) in previously normotensive women accompanied by one or more of the following new-onset conditions at or after 20 weeks of pregnancy: proteinuria; maternal organ dysfunction (acute kidney injury, liver involvement, neurological, or hematological complications); and uteroplacental dysfunction [1]. According to the current concept, early-onset (EO) preeclampsia develops before 34 weeks of gestation, and it could relate to insufficient implantation disorder with abnormal placentation in the pre-clinical phase [3]. Following the 20 weeks of gestation, the growth of the fetus accelerates, but the aberrant placenta cannot compensate it, which leads to clinical symptoms (hypertension and proteinuria) [4].

Numerous studies have investigated preeclampsia from the clinical aspects [5,6,7], but much fewer from the immunological point of view. Preeclampsia is accompanied by a systemic Th1 immune status caused by primarily pro-inflammatory factors (soluble fms-like tyrosine kinase 1 or placental growth factor) releasing from the hypoxic placenta [8]. Furthermore, the imbalance of the pro- and anti-inflammatory cytokine releasing could contribute to endothelial dysfunction and vascular abnormalities [9]. At the same time, significant changes can be observed in the maternal immune system in women diagnosed with preeclampsia. Studies reported that the ratio of the regulatory T cells (Treg) and Th17 subpopulations is decreased in preeclampsia compared to a healthy pregnancy [10,11]. Furthermore, it is well known that the proportion of decidual Treg cells is reduced in preeclamptic conditions [12]. A further study reported that the decreased number of decidual NK cells could be linked to the impaired trophoblast invasion, which results from placental hypoxia and could lead to preeclampsia and intrauterine growth restriction [13].

TIGIT (T-cell Ig and ITIM domain) was first described in 2009 by a bioinformatics study [14] to identify new genes related to T cell activation. This surface receptor belongs to the poliovirus receptor (PVR) family and mediates inhibitory signals following the interaction with the ligands CD155 and CD112. The presence of the TIGIT receptor has been proved on the surface of activated αβ-T cells [15], Tregs [16], NKT, and NK cells [17]. As an inhibitory immune checkpoint molecule, the TIGIT mediated signal down-regulates the effector function of T cells [18] and T cell receptor expression [19], and decreases NK cell cytotoxicity and cytokine production [17]. Similar to other checkpoint inhibitors, TIGIT was also involved in the anti-tumor target development. Currently, seven TIGIT-targeting therapeutics are in the early phase of clinical trials [20]. Interestingly the anti-TIGIT antibody with a combination of PD-1 blockade could improve the efficacy of the anti-tumor treatment representing a promising clinical therapeutic strategy [21].

CD226 (DNAM-1) is an activating receptor belonging to the immunoglobulin superfamily. CD226 is expressed on most immune cell surfaces, especially on T-, NK cells, and monocytes [22,23], and it has an important role in the induction of CD8+ T and NK cell-mediated immune responses after the ligation of CD155 and CD112 molecules [24]. As an activatory counterpart of TIGIT, CD226 competes for ligand binding [25].

Poliovirus receptor (PVR; CD155) and Nectin-2 (CD112) are related molecules and part of the Nectin family [26,27]. However, these ligand molecules are widely expressed on epithelial, endothelial cells, and hematopoietic cells at low levels [28], while high expression was published in several tumor cells [29,30,31]. In primary tumors, CD155 and CD112 have a potential role in tumor progression and migration [32] since interaction with TIGIT receptor expressed by lymphocytes and NK cells can inhibit the anti-tumor activity, including the maladaptation of granule releasing and IFN-γ production of these effector cells [33,34]. At the same time, the interaction between either CD155 or CD112 with CD226 on lymphocytes and NK cells can promote the cytotoxic activity of these effector cells against the tumors [27,35].

However, numerous studies, especially in tumor immunology, investigated TIGIT, CD226, CD112, and CD155 immune checkpoint molecules and their possible role in immunoregulation while much fewer papers have been published in the context of pregnancy; moreover, there is no published data related to EO preeclampsia.

## 2. Materials and Methods

### 2.1. Patients

A total of 37 pregnant women with the classic symptoms of preeclampsia (hypertension and proteinuria) were enrolled in this case-control study (Table 1). The diagnosis of the early-onset form of preeclampsia was based on the ISSHP definition, increased blood pressure (≥140 mmHg systolic or ≥90 mmHg diastolic on ≥2 separate occasions at least 4 h apart within a 24 h period) that occurred before the 34th week of gestation in women with previously normal blood pressure, accompanied by organ failure, such as significant proteinuria (≥30 mg/mol protein in 24-h urine collection in the absence of urinary tract infection). The blood sample was collected on the day of the diagnosis.

In total, 36 control participants with appropriately matched gestational age were involved from the Department of Obstetrics and Gynaecology, University of Pecs (Table 1). The control group consisted of healthy pregnant women without any substantial medical history, current or recent illnesses, or those taking medications. Further exclusion criteria were multiple gestations, preterm birth, primary hypertension, congenital malformations, angiopathy, diabetes mellitus, autoimmune disease, renal disorder, and intrapartum infection.

### 2.2. Sample Collection, PBMC Separation, and Cryopreservation

A heparinized venous blood sample was collected from all participants, and peripheral blood mononuclear cells (PBMC) were isolated on Ficoll-Paque density (GE Healthcare, Chicago, IL, USA) gradient. Separated cells were then washed in RPMI 1640 medium (Lonza, Basel, Switzerland), counted, and centrifuged again. Next, PBMC were resuspended in human serum containing 10% DMSO (Sigma-Aldrich, St. Louis, MO, USA) for cryoprotection. After that, cells were aliquoted in cryovials and stored in a −80 °C mechanical freezer. On the day of fluorescent cell labeling, cryovials were warmed up as quickly as possible in a 37 °C water bath and DMSO was washed out twice in RPMI 1640 medium.

### 2.3. Flow Cytometry

To block the Fc receptors expressed by the monocytes, Human TruStain FcX Blocking Solution (Biolegend, San Diego, CA, USA) was added to our thawed samples for 10 min before the flow cytometric staining procedure. Using fluorochrome-conjugated monoclonal antibody (Table 2) combinations, 106 PBMC were labeled for 30 min at room temperature in complete darkness. After a washing step, the cells were resuspended in 300 µL PBS containing 1% paraformaldehyde (PFA) and stored at 4 °C in complete darkness until FACS analysis. Flow cytometric analyses were performed with a BD FACS Canto II flow cytometer (BD Immunocytometry Systems, Erembodegem, Belgium) with the BD FACS Diva V6. software for data acquisition. Flow cytometric analysis data were performed with FCS Express V4 (De novo software, Pasadena, CA, USA).

### 2.4. Intracellular Staining

After surface labeling, cells were washed with PBS and fixed in 4% PFA for 10 min at room temperature in darkness. Next, the cells were washed with PBS and incubated with 1:10 diluted FACS Permeabilizing Solution 2 (BD Biosciences, Franklin Lakes, NJ, USA) for 10 min at room temperature in darkness. Following washing steps, the cells were incubated with FITC-conjugated anti-human granzyme B and PE-Cy7-conjugated anti-human perforin for 30 min at room temperature in complete darkness. Finally, the samples were washed with PBS, fixed with 1% PFA, and stored at 4 °C in the darkness until FACS analysis.

### 2.5. Statistical Analysis

The different expression of TIGIT, CD226 receptors, and CD112 and CD155 ligands in healthy or patients diagnosed with EO preeclampsia was statistically tested in R, version 4.0.5 [36], using Two-Samples t tests in case of a normal distribution or with the Mann–Whitney U test in case of non-normal distribution. Normality was tested with the Shapiro–Wilk test, individually for each dependent variable.

## 3. Results

### 3.1. Investigating the Percentage of Different Immune Cell Subpopulations in Healthy Pregnant and EO Preeclamptic Women

**Figure 1:**
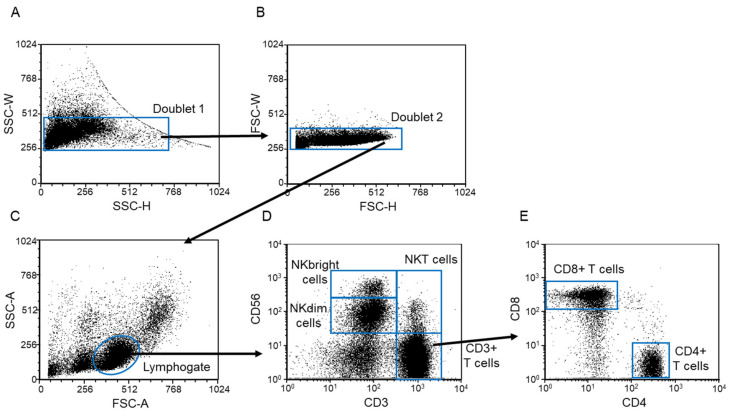
Gating strategy to detect lymphocyte subpopulations. Gating technique used to determine lymphocyte subpopulations from peripheral blood mononuclear cells. After a two-step doublet exclusion (**A**,**B**), the lymphocyte population was gated using FSC-A/SSC-A parameters (**C**). From the lymphogate CD3+ T-, NK-, NKdim-, NKbright-, NKT-, CD8+ T-, and CD4+ T cell subpopulations were detected (**D**,**E**).

Lymphocyte and monocyte subsets were determined based on the gating strategy (Figure 1 and Figure 7). Following the comparison of the isolated lymphocyte (CD4+ T, CD8+ T, Treg, NK, NKdim, NKbright, and NKT-like) and monocyte (classical, intermediate, and non-classical) subpopulations from peripheral blood, notable differences were not detected between the healthy and the EO preeclamptic cohort (Table 3).

The percentage of different immune cell subsets in the lymphogate (Figure 1C) and the monocyte gate (Figure 7G). The results were expressed as the mean values ± standard deviation of the mean (SD). Differences were considered significant when the values of *p* was equal to or less than 0.05.

### 3.2. Different Immune Checkpoint Receptor Expression by Immune Cell Subpopulations in Healthy Pregnancy and EO Preeclampsia

**Figure 2:**
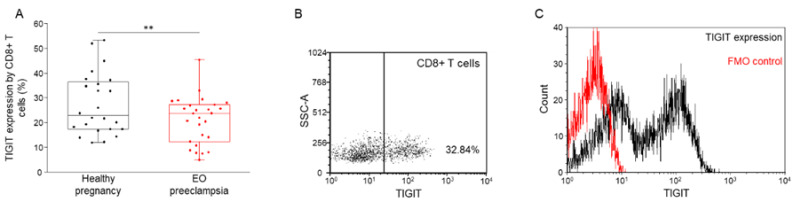
TIGIT expression by CD8+ cytotoxic T cells in women with EO preeclampsia and healthy pregnant women. The expression of TIGIT by CD8+ cytotoxic T cells in the peripheral blood of women with EO preeclampsia and healthy pregnant women (**A**). The solid bars represent medians of 24 and 27 determinations, respectively, the boxes indicate the interquartile ranges, and the lines show the most extreme observations. Differences were considered statistically significant for *p*-values < 0.05 **. Representative FACS plots show the expression of TIGIT surface marker by CD8+ T cells (**B**). To determine the positivity of the TIGIT receptor, fluorescent minus one (FMO) control was used (**C**).

**Figure 3:**
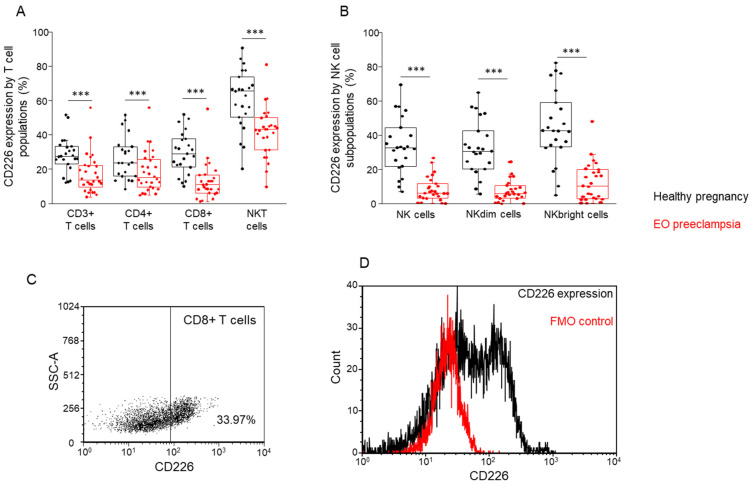
CD226 expression by the investigated T- and NK cell subpopulations in women with EO preeclampsia and healthy pregnant women. The expression of CD226 by different T- and NK cell subpopulations in the peripheral blood of women with early-onset preeclampsia and healthy pregnant women (**A**,**B**). The solid bars represent medians of 24 and 27 determinations, respectively, the boxes indicate the interquartile ranges, and the lines show the most extreme observations. Differences were considered statistically significant for *p*-values < 0.01 ***. Representative FACS plots show the expression of CD226 receptor by CD8+ T cells (**C**). To determine the positivity of the CD226 receptor, FMO control was used (**D**).

The surface expression of the TIGIT checkpoint inhibitor was determined using multicolor flow cytometry. Significantly decreased receptor expression was detected by CD8+ T cell subsets in EO preeclamptic patients compared to healthy pregnant women (Figure 2A). Similar to TIGIT, the expression level of the activating immune checkpoint receptor CD226 was also significantly lower by CD3+ T-, CD4+ T, CD8+ T, and NKT-like cells in women diagnosed with EO preeclampsia compared to the healthy pregnant cohort (Figure 3A). Measuring the CD226 expression, significantly decreased surface receptor expression level was detected by the NK-, NKdim-, and NKbright cell subpopulations in women with EO preeclampsia compared to the healthy pregnant control group (Figure 3B).

### 3.3. Comparing the Frequency of the TIGIT and CD226 Receptor-Positive and -Negative T Cell Subpopulations in Healthy Pregnancy and EO Preeclampsia

**Figure 4:**
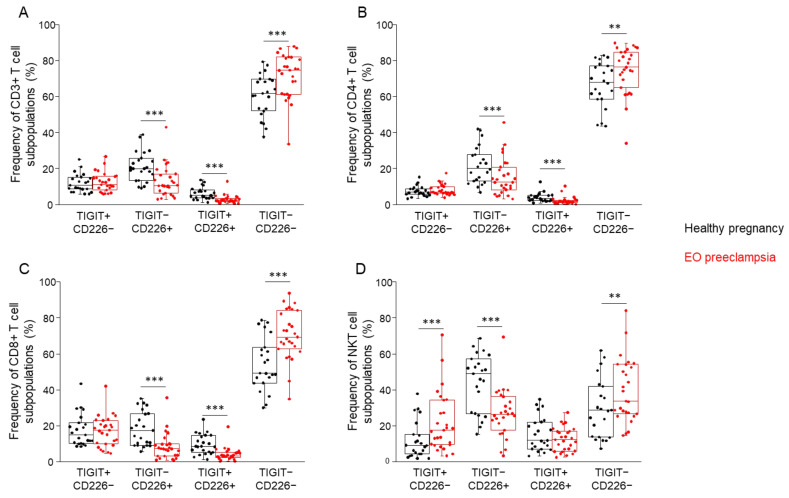
The frequency of the investigated T cell subpopulations is based on the presence or absence of TIGIT and CD226 receptors in women with EO preeclampsia and healthy pregnant women. The frequency of CD3+ T- (**A**), CD4+ T- (**B**), CD8+ T- (**C**), and NKT (**D**) cell subpopulations is based on the surface presence or absence of the TIGIT and CD226 receptors in women with EO preeclampsia and healthy pregnant women. The solid bars represent medians of 24 and 27 determinations, respectively, the boxes indicate the interquartile ranges, and the lines show the most extreme observations. Differences were considered statistically significant for *p*-values < 0.05 **, *p* < 0.01 ***.

After the surface receptor examination, the frequencies of the receptor-positive and receptor-negative cell subpopulations were determined. The double negative (TIGIT−/CD226−) subpopulations have the highest frequency in the case of CD3+ T-, CD4+ T-, and CD8+ T cells. Furthermore, in these cell populations significantly higher frequencies were detected in the EO preeclamptic cohort compared to the healthy cohort (Figure 4A–C). On the contrary, the double-positive (TIGIT+/CD226+) subpopulations have the lowest frequency in these T cell subsets; moreover, significantly lower frequencies were determined in the EO preeclamptic cohort compared to the healthy cohort (Figure 4A–C). We also detected significantly lower frequencies in TIGIT-/CD226+ cells in EO preeclamptic patients compared to healthy controls in the case of CD3+ T-, CD4+ T-, and CD8+ T cells (Figure 4A–C). Examination of the NKT cell subpopulation significantly elevated frequencies were measured in TIGIT+/CD226− and TIGIT−/CD226− subsets in EO preeclamptic cohort compared to healthy cohort; however, the frequency of the TIGIT−/CD226+ subpopulation was significantly decreased in EO preeclampsia (Figure 4D).

### 3.4. Comparing the Frequency of the TIGIT and CD226 Receptor-Positive and -Negative NK Cell Subpopulations in Healthy Pregnancy and EO Preeclampsia

**Figure 5:**
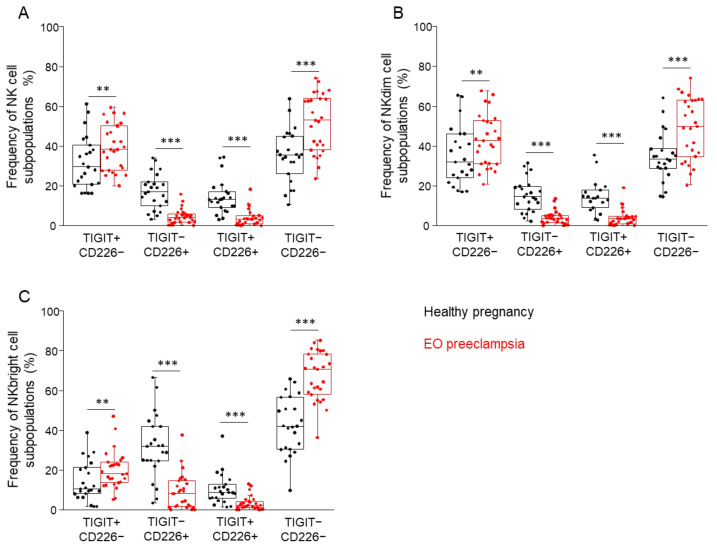
The frequency of the investigated NK cell subpopulations is based on the presence or absence of TIGIT and CD226 receptors in women with EO preeclampsia and healthy pregnant women. The frequency of NK- (**A**), NKdim- (**B**), and NKbright (**C**) cell subpopulations is based on the surface presence or absence of the TIGIT and CD226 receptors in women with EO preeclampsia and healthy pregnant women. The solid bars represent medians of 24 and 27 determinations, respectively, the boxes indicate the interquartile ranges, and the lines show the most extreme observations. Differences were considered statistically significant for *p*-values < 0.05 **, *p* < 0.01 ***.

Investigating the NK-, NKdim-, and NKbright cell populations, a similar trend was revealed regarding the frequencies of the receptor-positive and/or -negative subpopulations. The frequency was significantly higher in the case of the TIGIT+/CD226− and TIGIT−/CD226− cells in EO preeclamptic patients; at the same time, the examined frequency of the TIGIT−/CD226+ and TIGIT+/CD226+ cells was significantly lower compared to the healthy group (Figure 5A–C).

### 3.5. Determining Perforin and Granzyme B Content in Different Immune Cell Subpopulations in Healthy Pregnant and EO Preeclamptic Women

**Figure 6:**
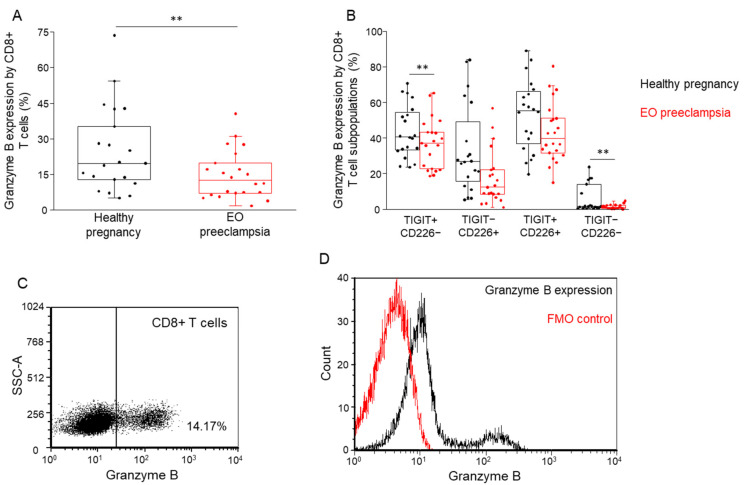
Granzyme B expression by the CD8+ T cell subpopulations in women with EO preeclampsia and healthy pregnant women. The intracellular expression of granzyme B molecule by CD8+ T cells in the peripheral blood of women with EO preeclampsia and healthy pregnant women (**A**). Granzyme B expression by CD8+ T cell subpopulations is based on the surface presence or absence of the TIGIT and CD226 receptors in women with EO preeclampsia and healthy pregnant women (**B**). The solid bars represent medians of 21 and 22 determinations, respectively, the boxes indicate the interquartile ranges, and the lines show the most extreme observations. Differences were considered statistically significant for *p*-values < 0.05 **. Representative FACS plots show the intracellular expression of granzyme B by CD8+ T cells (**C**). To determine the positivity of granzyme B, FMO control was used (**D**).

Intracellular perforin and granzyme B production of CD8+ T, NK, NKdim, NKbright, and NKT cells were measured. Perforin expression in healthy individuals was not different in the investigated cell populations compared to the preeclamptic group (data not shown). Furthermore, significantly reduced granzyme B content was observed in the case of CD8+ T cells in women diagnosed with EO preeclampsia compared to healthy women (Figure 6A). Investigating the granzyme B content of the receptor-positive and receptor-negative subpopulations, the intracellular expression of granzyme B was significantly decreased in TIGIT+/CD226− and TIGIT−/CD226− subsets; however, the two other examined subsets exhibit a similar trend (Figure 6B).

### 3.6. Investigating the Frequency of the Monocyte Subpopulations in Healthy Pregnant and EO Preeclamptic Women

**Figure 7:**
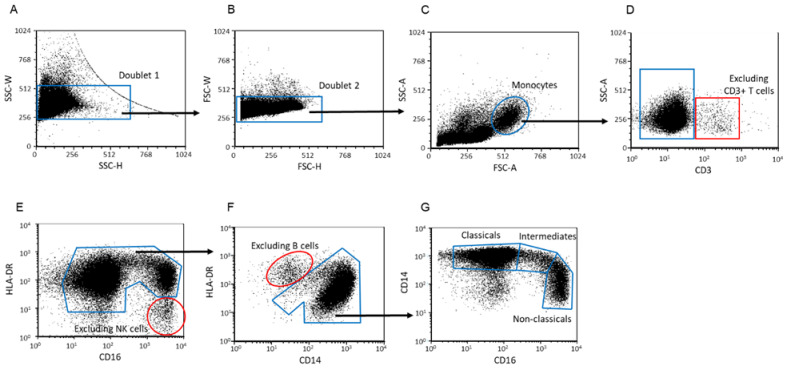
Gating strategy to detect monocyte subpopulations. The gating technique was used to determined monocyte subpopulations from peripheral blood mononuclear cells. After a two-step doublet exclusion (**A**,**B**), the monocyte population was gated using FSC-A/SSC-A parameters (**C**). From roughly gated monocytes, the CD3+ cell population was excluded (**D**). CD16 vs. HLA-DR dot plot: CD16+/HLA-DR− NK cells could exclude from the monocytes (**E**). CD14 vs. HLA-DR dot plot: HLA-DR high/CD14 low B cells could exclude from the monocytes (**F**). Using CD16 and CD14 markers, monocytes were gated based on their characteristic “┐” shape, and subpopulations were differentiated (**G**).

Using flow cytometric analyses, monocyte subpopulations were differentiated into classical, intermediate, and non-classical subsets according to Marimuthu et al. [37]. Briefly, after a doublet exclusion (Figure 7A,B), monocytes were roughly gated (Figure 7C) and excluded the CD3+ cells (Figure 7D). To exclude the possible gated NK cell, CD16 and HLD-DR markers were applied (Figure 7E). Furthermore, to exclude the possible gated B cells, CD14 and HLD-DR makers were used (Figure 7F), employing a CD16/CD14 dot plot to gate the selected monocyte subsets based on their characteristic “┐” shape (Figure 7G).

### 3.7. Comparing the CD112 and CD155 Immune Checkpoint Ligand Expression by the Investigated Monocyte and Lymphocyte Subpopulation in Healthy Pregnant and EO Preeclamptic Women

**Figure 8:**
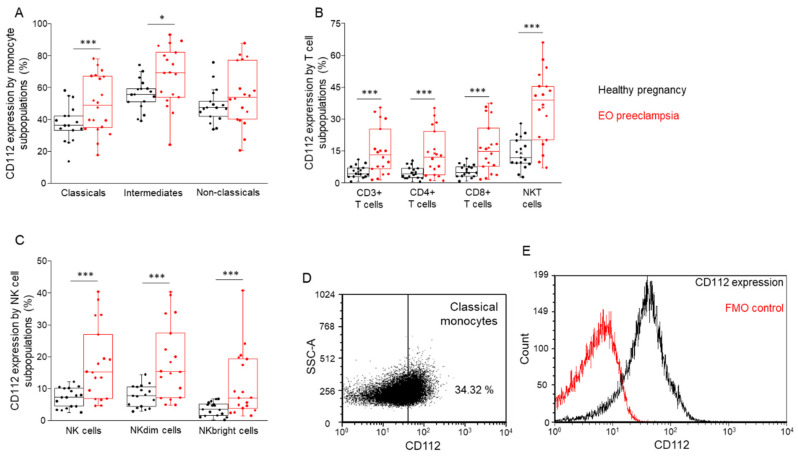
CD112 expression by the investigated immune cell subpopulations in women with EO preeclampsia and healthy pregnant women. The expression of CD112 by different monocyte (**A**), T cell (**B**), and NK cell (**C**) subpopulations in the peripheral blood of women with EO preeclampsia and healthy pregnant women. The solid bars represent medians of 17 and 20 determinations, respectively, the boxes indicate the interquartile ranges, and the lines show the most extreme observations. Differences were considered statistically significant for *p*-values < 0.05 *, *p* < 0.01 ***. Representative FACS plots show the expression of CD112 receptors by classical monocytes (**D**). To determine the positivity of the CD112 receptor, FMO control was used (**E**).

**Figure 9:**
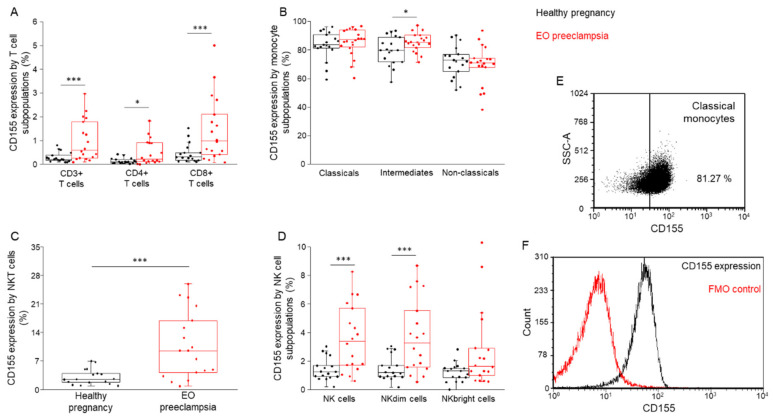
CD155 expression by the investigated immune cell subpopulations in women with EO preeclampsia and healthy pregnant women. The expression of CD155 by different monocyte (**A**), T cell (**B**), NKT cell (**C**), and NK cell (**D**) subpopulations in the peripheral blood of women with EO preeclampsia and healthy pregnant women. The solid bars represent medians of 17 and 20 determinations, respectively, the boxes indicate the interquartile ranges, and the lines show the most extreme observations. Differences were considered statistically significant for *p*-values < 0.05 *, *p* < 0.01 ***. Representative FACS plots show the expression of CD155 receptor by classical monocytes (**E**). To determine the positivity of the CD155 receptor, FMO control was used (**F**).

Following the characterization of the monocyte and lymphocyte subsets, surface immune checkpoint ligand expression was determined by flow cytometry. Investigating the presence of CD112 in the cell surface, significantly elevated ligand expression was measured in all monocytes except for non-classical cells (Figure 8A), T-cells, and NK cells (Figure 8B,C) in the EO preeclampsia cohort compared to the healthy group. Similarly, increased CD115 surface expression levels were detected in almost all examined cell populations except non-classical monocytes and NKbright cells (Figure 9A–D).

## 4. Discussion

Pregnancy-related hypertensive disorders affect up to 10% of pregnancies globally and could associate with 8–10% of all preterm births [38]. Preeclampsia is a severe form of hypertensive pregnancy disorder, accompanied by organ dysfunction, neurological symptoms, or uteroplacental dysfunction [12]. Since the only potential therapy for preeclampsia is iatrogenic preterm birth, it is crucial to monitor high-risk pregnancies and apply an early prediction model for prevention and future treatment.

During pregnancy, a unique immunological condition develops in the maternal immune system to tolerate the presence of the semi-allogenic fetus. This immunological tolerance is essential for the healthy development of the fetus. At the same time, its absence has serious consequences. It is proven that in preeclampsia, the immunological balance between the mother and the fetus is changed, which affects innate and adaptive immunity. Our knowledge is much less extensive about the preeclampsia-related immunoregulatory mechanisms, especially about immune checkpoint pathways. Moreover, there is no available information about the TIGIT, CD226, CD112, or CD155 immune checkpoint molecules in any context of preeclampsia.

Therefore, we first determined and compared the surface expression of the two immune checkpoint receptors, TIGIT and CD226, on different immune cell subpopulations. However, significantly reduced inhibitory TIGIT expression was detected only in the case of the preeclamptic CD8+ T cells, which might be a part of the Th1 predominance during EO preeclampsia at the same time, the expression of the activatory counterpart CD226 also exhibited a decreased pattern in preeclampsia, not only the CD8+ T cells, but also in all investigated cell population. The general reduction in the CD226 receptor expression might be a compensatory mechanism to regulate the more pronounced Th1 response, but also to indicate the maladaptation of the maternal immune regulation. Following that, we investigated the receptor-positive and/or receptor-negative immune cell subsets. Interestingly, but related to the previously mentioned expression results, the ratio of CD226 expressed NK cells is significantly decreased while the ratio of CD226 negative NK cells is significantly increased in preeclamptic women independently of the presence of TIGIT receptor. This pattern is quite similar in the case of the investigated T cells; therefore, the presence of the CD226 activatory receptor could play a role in disturbing immune responses related to EO preeclampsia.

However, we did not find any difference in the intracellular perforin content, while a reduced granzyme B expression was detected by CD8+ T cell population in EO preeclamptic women. Since both the investigated receptor expression was decreased by this population after further analyses, we found a significantly reduced granzyme B content only in the CD226 receptor-negative CD8+ T cells, independently of the TIGIT expression. Since CD226 is constitutively expressed on the surface of the majority of T lymphocytes and NK cells [22], the reduction in the surface presence could result from the Th1 predominance during EO preeclampsia. Furthermore, it is possible that the decreased presence of the activatory receptor led to the reduced granzyme B content since there is a smaller chance to interact with the ligand molecules.

Following the investigation of the receptor molecules, we examined the other side of the checkpoint pathway; therefore, according to a recent publication [37], we identified three different monocyte subpopulations and determined their CD122 and CD155 ligand expression. Except for the non-classical monocyte subpopulation, a general elevation was detected regarding the surface expression of CD122, which might support our theory that a compensatory mechanism tries to regulate the Th1 predominance since in the decreased presence of activatory CD226 receptor, the CD122 ligand is more likely to bind the inhibitory TIGIT receptor. This fact—that TIGIT disrupts CD226 co-stimulation and can bind to the CD122 with a higher affinity [14]—is further supporting our hypothesis. However, an increased expression of another ligand CD155 has been detected only in the intermediate monocytes and other cell subsets from the lymhpogate. The CD155/TIGIT interaction is also possible since TIGIT has a higher affinity to CD155 than CD112 [39]. The fact that PVR has a higher affinity to TIGIT than CD226 emphasizes the dominance of inhibitory signaling over activatory signals. Moreover, the decreased surface expression of CD226 receptor further supports this theory in the case of EO preeclampsia. Furthermore, a publication showed that after the PVR-TIGIT connection, an elevated IL-10 and decreased IL-12p40 production by human dendritic cells (DCs) was revealed, which can contribute to further downregulation of the T cell activation [14]. This mechanism might have a role in letting the immune system react to the Th1 predominance during EO preeclampsia. Since the systemic immune response is probably a reflection of the local immune alterations observed at the materno-fetal interface (MFI), recent studies showed the importance of immune checkpoint molecules present locally [40,41,42]. There is no available data regarding the potential role of TIGIT/CD226/CD112/CD155 molecules at the MFI, but it needs further investigation.

## 5. Conclusions

Overall, the role of the immune checkpoint molecules and pathways is to maintain the immunological balance between the pro- and anti-inflammatory responses/reactions. During pregnancy, a particular immunological environment develops to tolerate and, at the same time, protect the fetus and the mother from certain infections. Our previous works demonstrate the potential role of immune checkpoint molecules [43,44] in feto–maternal tolerance and their different expression pattern in EO preeclampsia [45,46]. We assume that immune checkpoint molecules, including TIGIT/CD226/CD112/CD155, have an essential role in the regulation of the Th1 predominance, which is initiated by intrinsic factors released by the poorly developed placenta during EO preeclampsia.

## Figures and Tables

**Table 1:** Patients’ demographic and gynecological characteristics.

	Healthy Pregnant Women	Early-Onset Preeclamptic Patients
No. of patients	36	37
Age (years)	32.25 (27–37)	29.71 (18–43)
Gestational age at birth (weeks)	39.11 ± 0.99	30.96 ± 3.14 *
Gestational age at sampling (weeks)	32.07 ± 3.92	30.00 ± 2.51
Birth weight (g)	3501.07 ± 368.36	1396.67 ± 602.82 *

The results were expressed as the mean values ± standard deviation of the mean (SD). Statistical comparisons were made using the independent sample t-tests. * *p* < 0.01 vs. healthy pregnant women.

**Table 2:** Fluorochrome conjugated monoclonal antibodies used in the study.

Antigen	Format	Clone	Isotype	Company	CAT
CD112	PE	R2.525	Mouse IgG1, κ	BD Biosciences	551057
CD14	FITC	M5E2	Mouse IgG2a, κ	BD Biosciences	555397
CD155	APC	SKII.4	Mouse IgG1, κ	Biolegend	337618
CD16	PerCp-Cy5.5	3G8	Mouse BALB/c x DBA/2, κ	BD Biosciences	560717
CD3	BV510	UCHT1	Mouse BALB/c IgG1, κ	BD Biosciences	563109
CD4	FITC	RPA-T4	Mouse IgG1, κ	BD Biosciences	555346
CD8	APC-H7	SK1	Mouse BALB/c IgG1, κ	BD Biosciences	560179
CD56	PerCp Cy5.5	B159	Mouse IgG1, κ	BD Biosciences	560842
CD56	APC	B159	Mouse IgG1, κ	BD Biosciences	555518
CD226	BV421	DX11	Mouse BALB/c IgG1, κ	BD Biosciences	742493
Granzyme B	FITC	GB11	Mouse BALB/c IgG1, κ	BD Biosciences	560211
HLA-DR	APC-H7	G46-6	Mouse IgG2a, κ	BD Biosciences	561358
NKG2D	PE-Cy7	1D11	Mouse RBF/DnJ IgG1, κ	BD Biosciences	562365
Perforin	PE-Cy7	dG9	Mouse IgG2b, κ	Biolegend	308126
TIGIT	PE	A1553G	Mouse IgG2a, κ	Biolegend	372704

**Table 3:** Phenotype analysis of peripheral blood mononuclear cells in 3rd-trimester healthy pregnant women and women with EO preeclampsia.

	Gate	Healthy Pregnant Women	Early-Onset Preeclamptic Patients	*p*-Values
CD3+ T cells	lymhogate	65.84 ± 9.67	62.24 ± 8.70	NS
CD4+ T cells	lymhogate	36.32 ± 9.75	33.56 ± 9.64	NS
CD4+ T cells in CD3+ T cells	lymhogate	57.35 ± 9.59	53.19 ± 11.65	NS
CD8+ T cells	lymhogate	22.30 ± 7.66	24.46 ± 7.34	NS
CD8+ T cells in CD3+ T cells	lymhogate lymhogate	35.25 ± 9.99	39.20 ± 10.60	NS
NK cells	lymhogate	14.20 ± 8.34	15.64 ± 6.63	NS
NKdim cells	lymhogate	11.65 ± 7.62	13.57 ± 5.95	NS
NKbright cells	lymhogate	2.52 ± 1.40	2.00 ± 1.29	NS
NKT-like cells	lymhogate	6.73 ± 4.47	4.87 ± 3.97	NS
Classical monocytes	monocyte	93.92 ± 2.52	92.23 ± 6.80	NS
Intermediate monocytes	monocyte	2.47 ± 1.13	3.07 ± 2.95	NS
Non-classical monocytes	monocyte	3.99 ± 1.77	3.87 ± 3.99	NS

## Data Availability

The data presented in this study are available on request from the corresponding author. The data are not publicly available as the participants gave permission to learn and handle their personal and research generated data only to researchers involved in this study.
